# SPR spectroscopic analysis of myosin binding to wild type and mutant UNC45B

**DOI:** 10.17912/micropub.biology.001131

**Published:** 2024-02-05

**Authors:** Silvana Valdebenito, Eliseo Eugenin, Andres Oberhauser

**Affiliations:** 1 The University of Texas Medical Branch at Galveston, Galveston, Texas, United States

## Abstract

UNC45B is a multidomain molecular chaperone that is essential for the proper folding and function of myosin. It has previously been demonstrated that the UCS domain is responsible for the chaperoning function of UNC45B and that removing its client-binding loop leads to a significant change in its solution conformation and a reduced chaperoning function. Here, we report the direct quantification of affinities of myosin binding to wild type and mutant UNC45B using surface plasmon resonance (SPR) spectroscopy. We found that deletion of the client-binding loop in UNC45B resulted in a dramatic decrease in myosin affinity.

**Figure 1. SPR spectroscopic analysis of myosin binding to wild type and mutant UNC45B:**
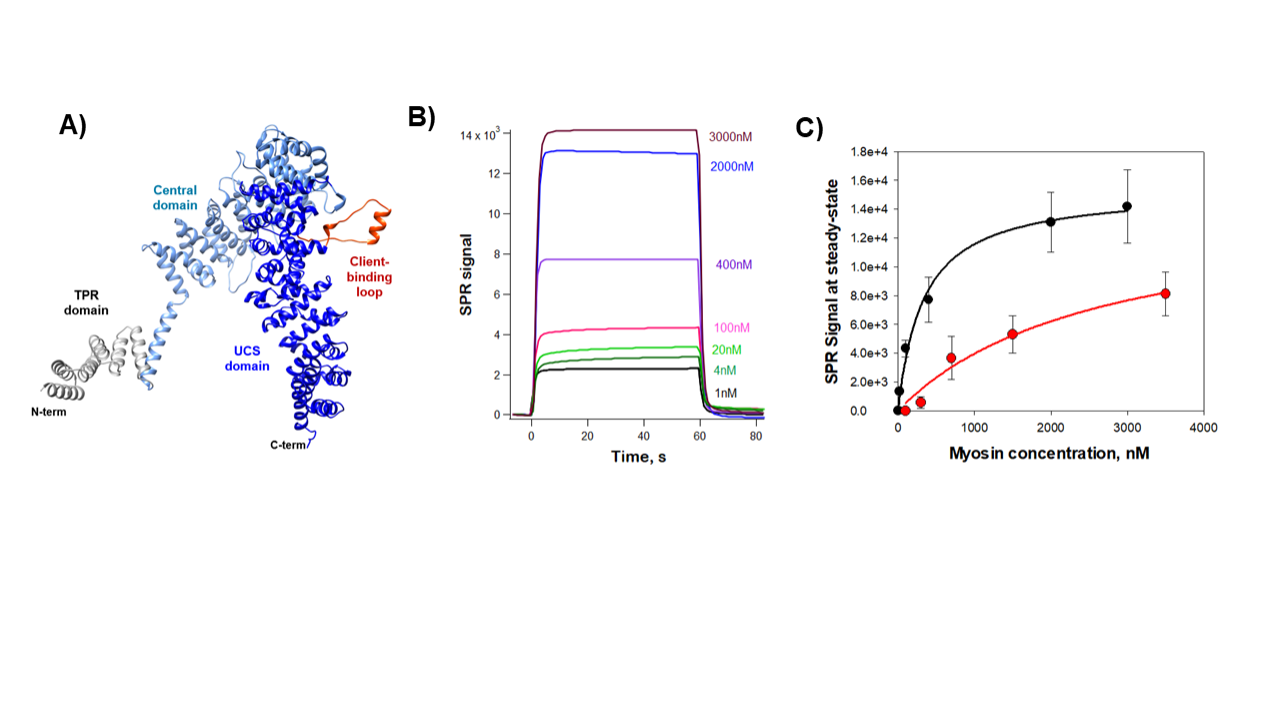
**A)** AlphaFold model of human UNC45B protein. The TPR, Central and UCS domain are depicted in grey, cyan and blue, respectively. The client-binding is shown in red. **B)** Sensorgrams (or binding curves) of myosin binding immobilized UNC45B as a function of time; myosin was injected at the indicated concentrations. **C)** Determination of the dissociation constant using a simple 1:1 one-site saturation ligand binding model (Y = Bmax*X)/(Kd + X); where Y is the SPR signal at steady-state and X is the ligand concentration). The estimated Kd’s are 0.33 ± 0.06 μM (n=3) for wild-type UNC-45B and 2.63 ± 1.03 μM (n=3) for the loop deletion mutant.

## Description

The arrangement of not just the thick and thin filaments, but numerous other proteins into the exacting arrangement of the semi-crystalline lattice making up the sarcomere is essential for muscle contractile function. This process is partially autonomous, an intrinsic property of its component proteins. However, the assembly of a functional sarcomere requires molecular chaperones (Crawford and Horowits 2011) . These serve to prevent aggregation of unfolded intermediates and also, according to recent evidence, to help assemble the sarcomere (Chow et al. 2002) . Amongst the sarcomeric chaperone proteins known to be involved in the folding of myosin is UNC45 (Chow et al. 2002; Srikakulam, Liu, and Winkelmann 2008; Lee, Melkani, and Bernstein 2014; Smith et al. 2014; Pokrzywa and Hoppe 2013) . Unique characteristics of UNC45 include its ability to recognize both folded and misfolded myosin proteins (Barral et al. 2002; Gazda et al. 2013; Landsverk et al. 2007a; Lee, Melkani, and Bernstein 2014; Melkani et al. 2011; Moncrief et al. 2021) . UNC45 is made up of three domains (Barral et al. 1998; Hellerschmied and Clausen 2014; Hutagalung et al. 2002; Landsverk et al. 2007b); **Figure 1A** . The canonical UCS domain, named for the UNC45/Cro1/She4p family of proteins, is responsible for the chaperone functionality and the interaction with myosin (Ni et al. 2011; Bujalowski, Nicholls, and Oberhauser 2014a; Barral et al. 1998; Barral et al. 2002; Bujalowski, Nicholls, and Oberhauser 2014b). The Central domain acts as an inhibitor of the myosin power stroke through a mechanism that allows ATP turnover (Bujalowski et al. 2018) . The TPR domain is known to allow interactions with other molecular chaperones including Hsp90 via the TPR binding region of the C-terminus of that protein (Barral et al. 2002) . Both the Central and UCS domains independently bind to myosin *in vitro* and *in vivo* (Lord, Sladewski, and Pollard 2008; Lord and Pollard 2004; Bujalowski, Nicholls, and Oberhauser 2014b). However, only the UCS domain displays chaperone activity on the myosin head, preventing thermal aggregation of unfolding intermediates and maintaining mechanically unfolded intermediates in folding competent states (Bujalowski, Nicholls, and Oberhauser 2014b).

An important myosin-interacting region of UCS proteins is the myosin-binding loop (601N---D630); colored in red in Figure 1A . This loop is predicted to be highly flexible based on molecular dynamic simulations, limited proteolysis, and MALDI mass spectrometry analysis (Bujalowski et al. 2015) . This correlates well with a mobile loop with minimal electron density conserved in the UNC45 structures of *C. elegans* and *D. melanogaster* (Gazda et al. 2013; Lee et al. 2011) , which further proved susceptible to trypsin digestion in *Drosophila* (Lee et al. 2011) and its deletion abolished myosin binding in immunoprecipitation experiments (Gazda et al. 2013) . We recently found that removing the myosin-binding loop altered the secondary structure of the UCS domain (by decreasing the α-helix content), leading to a significant change in its solution conformation and a reduced chaperoning function (Gaziova et al. 2020) .

However, it is not known how these conformational changes that occur when the UNC45B client-binding loop is deleted translate into changes in binding affinities to its myosin. In order to address this question, we directly quantified the interaction between UNC45B and myosin using surface plasmon resonance (SPR). SPR technology has proven particularly useful in monitoring biomolecular binding interactions with high sensitivity and accuracy using very small sample protein concentration (nM) and small volumes (microliters) (Nguyen et al. 2015) . We quantified the affinity between recombinant UNC45B immobilized on a Ni ^2+-^ NTA chip via its His-tag and purified full-length myosin. We used 100 μl of a 100 nM UNC45B solution and increasing concentrations of myosin (1 up to 3500 nM, or 20ng-60 μg in 100 μl), to measure the Sensorgrams (or binding curves) of myosin binding immobilized UNC45B as a function of time; **Figure 1B** . We then plotted the SPR signal at steady-state as a function of the myosin concentration and fitted the data to a 1:1 one-site saturation ligand binding model ( **Figure 1C** ). The binding shows a smooth (hyperbolic) saturable dependence of the concentration of myosin. Using SPR, we measure a dissociation constant, Kd, of 0.3 μM, a value that is 8.5-fold lower to that estimated by previous in vitro fluorescence experiments (Kd = 2.8 μM; (Bujalowski, Nicholls, and Oberhauser 2014c)). This apparent discrepancy is likely because the environmentally sensitive fluorophore BADAN used in these experiments does not directly measure protein-protein binding interactions but rather changes in the effective solvent polarity (Koehorst, Spruijt, and Hemminga 2008) . Our data clearly show that the deletion of the client-binding loop results in a dramatic decrease in the affinity by about 8-fold (estimated Kd = 2.6 μM). Hence, our results confirm the hypothesis that the client-binding loop plays a key role to recognize and bind myosin. Important future SPR experiments to perform are: 1) to elucidate the mechanisms of how the myosin-binding loop and/or other UCS regions are able to recognize both folded and misfolded myosins and 2) to better understand how these molecular recognitions are regulated by other proteins (e.g. Hsp90).

## Methods

**Mutagenesis, Protein Expression, and Purification of proteins**

UNC45B and the loop deletion mutant (residues 600-630) from Homo sapiens (gene identifier: UNC45B, NCBI Gene ID: 146862) were codon optimized for expression in *Escherichia coli* , synthesized (GenScript, Piscataway, NJ), and subcloned into a pET-28 vector (EMD Millipore, Billerica, MA). The UNC45B constructs were expressed and purified using published methods (Gaziova et al. 2020; Bujalowski, Nicholls, and Oberhauser 2014c; Moncrief et al. 2021) ). Briefly, recombinant protein expression was induced in BL21 DE3 when optical density (OD600) reached 1-1.2 with 0.02 mM IPTG for 16 h at 15 ^o^ C. Harvested cells were resuspended in lysis buffer (50 mM Tris-HCl, 50 mM NaCl, 40 mM imidazole, 2 mM TCEP, 10% glycerol, pH 8.0) containing 1 mg/ml lysozyme and incubated for 30 min at RT. Insoluble material was removed by centrifugation at 30,000 x g for 30 min at 4 ^o^ C and the supernatant was filtered through a 0.45 µm syringe filter afterward. The supernatant was incubated with lysis buffer equilibrated HisPur Ni-NTA Resin (Thermo Scientific) for 1 hour at 4 °C. Resin with bound proteins was collected by centrifugation at low force (700 x g for 2-3 min), washed by 4 washes with ice-cold wash buffer (50 mM Tris-HCl, 50 mM NaCl, 60 mM imidazole, 2 mM TCEP, 10% glycerol, pH 8.0) and with last wash applied to the column cartridge. His-tagged recombinant proteins were eluted in 1 ml fractions with elution buffer (50 mM Tris-HCl, 50 mM NaCl, 250 mM imidazole, 2 mM TCEP, 10% glycerol, pH 8.0) and immediately 4 µl of EDTA pH 8.0 were added. Eluted fractions containing a significant portion of recombinant protein were pooled and dialyzed against storage buffer (50 mM Tris-HCl, 50 mM NaCl, 2 mM EDTA, 2mM TCEP, 10% glycerol, pH 8.0) overnight at 4°C. Proteins were concentrated to 20-40 µM, flash frozen, and long-term stored at -80°C. Myosin was purified from rabbit skeletal muscle using published methods (Margossian and Lowey 1982) . MWs and purity of all proteins were evaluated by SDS-PAGE.

**Measuring Myosin and chaperone affinity using Surface Plasma Resonance (SPR)**

We quantified the affinity between the UNC45B constructs (wild-type and mutant) immobilized on a Ni ^2+-^ NTA chip via their His-tags and myosin II using a BIAcore X100 (Cytiva) instrument, designed for biomolecular interaction analysis in real-time. We used this sensor chip with a pre-immobilized nitrilotriacetic acid (Sensor Chip NTA from Cytiva) to capture the histidine-tagged UNC45B constructs via Ni ^+2^ /NTA chelation. The running buffer for all the experiments was HBS-P (10 mM HEPES, 150 mM NaCl, 0.005% Tween20, pH 7.4) plus 50 μM EDTA. All buffers were filtered and degassed before use. The NTA sensor chip was activated with a saturated solution of 0.5 mM of NiCl _2_ for 60 and then followed by 3 mM EDTA solution to remove any traces of Nickel. The ligands (HisTagged UNC45B constructs, 0.2 μM) were injected over the Nickel activated surface for the 60s and then serial dilutions of the myosin analyte (1 up to 3500 nM of myosin) were injected over ligand surfaces at a flow rate of 5 μl/min. Sensorgrams were recorded and normalized to a baseline. Equivalent volumes of each protein dilution were also injected over a mock, nonprotein, blocked surface to serve as blank sensorgrams for subtraction of refractive index background. We estimated of the dissociation constant by plotting the SPR signal at steady-state as a function of the myosin concentration and fitting using a simple 1:1 one-site saturation ligand binding model (Y = Bmax*X)/(Kd + X); where Y is the SPR signal and X is the ligand concentration) implemented in Sigmaplot 14. Each determination was repeated three times. All the experiments were performed at 20 ^o^ C.

**Protein structure modeling**

For the human UNC45B protein structure modeling, we used the AlphaFold predicted model (Jumper et al. 2021) . Molecular graphics were generated by using Chimera (Pettersen et al. 2004) .
